# ^1^H, ^13^C and ^15^N resonance assignments of human BASP1

**DOI:** 10.1007/s12104-012-9436-4

**Published:** 2012-11-20

**Authors:** Leonhard Geist, Anna Zawadzka-Kazimierczuk, Saurabh Saxena, Szymon Żerko, Wiktor Koźmiński, Robert Konrat

**Affiliations:** 1 Department of Computational and Structural Biology, Max F. Perutz Laboratories, University of Vienna, Campus Vienna Biocenter 5, 1030 Vienna, Austria; 2 Faculty of Chemistry, University of Warsaw, Pasteura 1, 02-093 Warsaw, Poland

**Keywords:** BASP1, NMR signal assignment, Intrinsically disordered protein, Myc oncogene, WT1

## Abstract

Brain acid-soluble protein 1 (BASP1, CAP-23, NAP-22) appears to be implicated in diverse cellular processes. An N-terminally myristoylated form of BASP1 has been discovered to participate in the regulation of actin cytoskeleton dynamics in neurons, whereas non-myristoylated nuclear BASP1 acts as co-suppressor of the potent transcription regulator WT1 (Wilms’ Tumor suppressor protein 1). Here we report NMR chemical shift assignment of recombinant human BASP1 fused to an N-terminal cleavable His6-tag.

## Biological context

The homologues of human BASP1 were first identified as the brain-specific proteins CAP-23 (cortical cytoskeleton-associated protein) in chicken brain (Widmer and Caroni 1990) and its rat homologue NAP-22 (neuron-specific acidic protein; Maekawa et al. 1993). Human BASP1 was originally isolated from neuronal cells (Mosevitsky 2005). Interestingly it appears to fulfill quite diverse tasks in the cell. N-myristoylated BASP1 has been described to be involved in neurite outgrowth and plasma membrane organization (Korshunova et al. 2008). It is able to interact with the inner leaflet of the plasma membrane via its myristoyl-anchor and sequesters Phosphatidyl-inositol-4,5-diphosphate (PIP2) into lipid rafts (Epand et al. 2004; Shaw et al. 2006). Recently it has been shown that liposomes containing anionic phospholipids induce oligomerization of BASP1. Interaction with calmodulin is followed by dissociation of BASP1 from the membrane and disruption of the oligomers (Zakharov and Mosevitsky 2010). Additionally, BASP1 is under the control of protein kinase C (PKC), which phosphorylates BASP1 at Ser5. It is hypothesized that phosphorylation leads to the disruption of the interaction of the N-terminal positive effector domain of BASP1 with anionic phospholipids (Laux et al. 2000).

Furthermore, non-myristoylated BASP1 appears to influence transcription regulation in the nucleus, greatly affecting the differentiation pathway of a cell. It has been discovered as a co-suppressor of WT1 function (Wilms’ Tumor suppressor protein 1) exerting its function by interacting with an N-terminal suppression domain of WT1 (Carpenter et al. 2004; Green et al. 2009). WT1 itself is a potent transcriptional regulator that activates or represses target genes including those for growth factors and regulators of cell division (Wagner and Roberts 2004). Aberrant expression of WT1 is associated with several childhood and adult cancers (Rivera and Haber 2005; Yang et al. 2007). Additionally, a recent study discovered BASP1 to be downregulated in v-Myc-transformed chicken fibroblasts. Strikingly, ectopic expression of BASP1 renders fibroblasts resistant to subsequent cell transformation by v-Myc and it has been shown that the inhibition of v-Myc-induced cell transformation by BASP1 affects the transcriptional regulation of Myc target genes (Hartl et al. 2009). Other findings, reporting the frequent down-regulation of BASP1 expression in ALL (acute lymphocytic leukaemia) and CLL (chronic lymphocytic leukaemia) (Yeoh et al. 2002; Wang et al. 2004), as well as apoptosis-induced cleavage of BASP1 and its subsequent translocation to the cytoplasm (Ohsawa et al. 2008), again highlight the importance of BASP1 in transcription regulation.

To provide molecular information about this potential tumour suppressor protein we have started the NMR structure determination of recombinant human BASP1. The near complete chemical shift assignment reveals that BASP1 belongs to the class of intrinsically disordered proteins.

## Methods and results

### Protein expression and purification

The coding region for hBASP1 (human BASP1) was amplified by PCR from the mammalian expression vector Flag-hBASP1-pTKX3 (Ohsawa et al. 2008) introducing a 5′ NcoI and 3′ NotI site. Subsequently the fragment was inserted in-frame into the NcoI and NotI sites of the bacterial expression vector pET-M11 (Pinotsis et al. 2006), yielding pET-M11-hBASP1, encoding hBASP1 fused to an N-terminal His6-tag plus the TEV-cleavage site (H6-hBASP1). ^15^N/^13^C labeled H6-hBASP1 was expressed in the *E. coli* strain Rosetta(DE3)pLysS following a new expression protocol for efficient isotopic labeling of recombinant proteins using a fourfold cell concentration in isotopically labeled minimal medium (Marley et al. 2001). The cells were collected after 4 h of expression at 37 °C by centrifugation at 5,000 rpm for 15 min and resuspended in 40 ml of ice-cold lysis buffer (20 mM Na_x_H_(3−x)_PO_4_, 50 mM NaCl, 10 mM imidazole, pH 7.2) per liter of the original bacterial culture. Bacteria were lysed by passing through a French press, and the cell lysate was cleared by centrifugation at 18,000 rpm for 20 min. The supernatant containing the soluble protein fraction was loaded onto a Ni^2+^ loaded HiTrap 5 ml affinity column (GE Healthcare), washed with 2 column volumes of high salt buffer (20 mM Na_x_H_(3−x)_PO_4_, 1.5 M NaCl, 10 mM imidazole, pH 7.2) and eluted with high imidazole buffer (20 mM Na_x_H_(3−x)_PO_4_, 50 mM NaCl, 0.5 M imidazole, pH 7.2) using a linear gradient of 15 column volumes. The H6-hBASP1 containing fractions were collected and the buffer was exchanged by 4 steps of concentration in an Amicon Ultra-15 centrifugal filter device 10 K NMWL (Amicon) and subsequent dilution in target buffer (20 mM Na_x_H_(3−x)_PO_4_, 50 mM NaCl, pH 6.0). NMR samples contain 1.5 mM uniformly ^15^N/^13^C labeled protein in 20 mM sodium phosphate (pH 6.0, in 90 % H_2_O and 10 % D_2_O), 50 mM NaCl and 0.2 % sodium azide.

### NMR experiments

All spectra were acquired at 298 K on an Agilent Direct Drive 700 MHz spectrometer using the standard 5 mm ^1^H–^13^C–^15^N triple-resonance probe head.

The backbone ^1^H, ^13^C and ^15^N resonances were assigned using sparse random sampling of indirectly detected time domains, in order to increase resolution. A 3D HNCO experiment was used as a base spectrum for SMFT (Sparse Multidimensional Fourier Transform) processing of higher dimensionality experiments (Kazimierczuk et al. 2010). Backbone assignment was achieved using 5D HN(CA)CONH (Kazimierczuk et al. 2010), (HACA)CON(CA)CONH (Zawadzka-Kazimierczuk et al. 2012b), (H)NCO(NCA)CONH (Zawadzka-Kazimierczuk et al. 2012b) and HNCOCACB (Zawadzka-Kazimierczuk et al. 2012b) experiments. Side-chain assignments were obtained using 5D HabCabCONH (Kazimierczuk et al. 2010), and H(CC-tocsy)CONH (Kazimierczuk et al. 2009) experiments.

All NMR data sets were processed by multidimensional Fourier transformation using the home written software package (http://nmr700.chem.uw.edu.pl/formularz.html). The resonance assignment was performed using the TSAR program (Zawadzka-Kazimierczuk et al. 2012a). The input data for TSAR was prepared using Sparky software (Goddard and Kneller 2002). Table 1 shows the maximum evolution times and spectral width used for the acquisition of the spectra.

**Table 1:** Maximum evolution times (tmax, ms) and spectral width (sw, kHz) used for acquisition of spectra for H6-hBASP1

	3D HNCO	4D HabCabCONH	5D HNCOCACB	5D HN(CA)CONH	5D H(CC-tocsy)CONH	5D (H)NCO(NCA)CONH	5D (HACA)CON(CA)CONH
Number of points	700	550	550	550	650	610	610
Experiment duration (hours)	4	13	13	13	17	14	14
sw_1_	2	4	14	6	8	2.5	3.8
sw_2_	2.5	14	14	2.5	18	2	2
sw_3_		2	2	2	2	2	2
sw_4_		2.5	2.5	2.5	2.5	2.5	3.8
*t*_1_^max^	100	20	10	25	10	50	50
*t*_2_^max^	150	7.1	10	50	10	45	45
*t*_3_^max^		45	45	50	45	45	45
*t*_4_^max^		75	75	75	75	75	75
Sampling density versus conventional	9.3 × 10^−3^	4.1 × 10^−6^	1.7 × 10^−6^	1.6 × 10^−6^	2.7 × 10^−6^	3.2 × 10^−8^	1.4 × 10^−7^

### Extent of assignment and data deposition

**Fig. 1:**
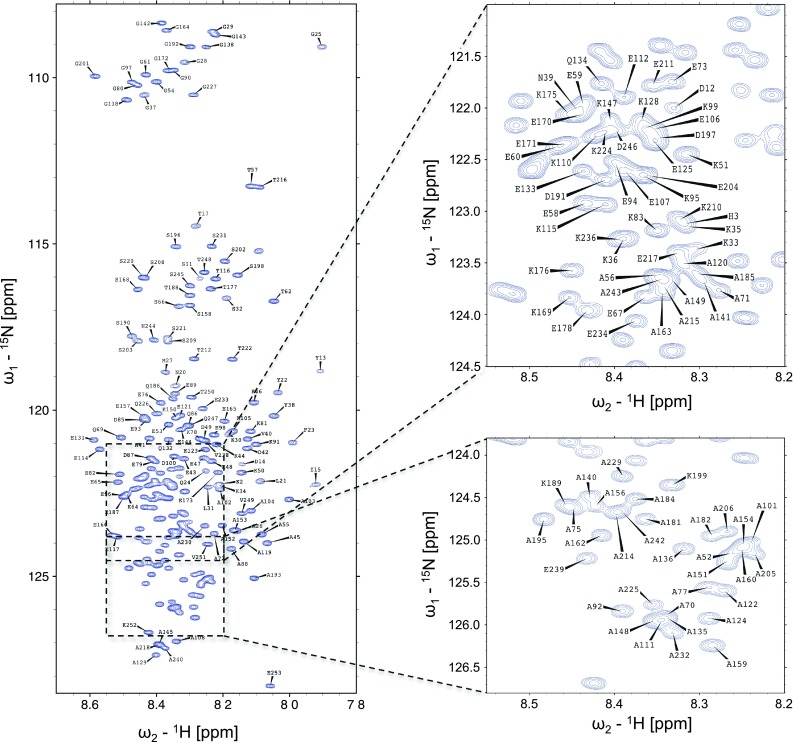
^1^H–^15^N HSQC spectrum of H6-hBASP1 at pH 6 and 298 K. Assignments of backbone amides are labeled in *single letter* amino acid code and residue number (His6-tag: 1–26; hBASP1: 27–253)

**Fig. 2:**
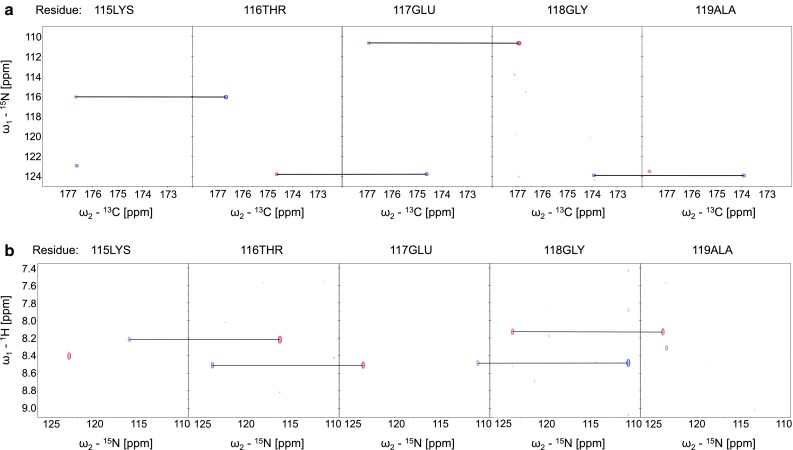
2D spectral planes for consecutive amino acids in H6-hBASP1 obtained by SMFT processing of the 5D randomly sampled signal. 2D cross-sections of **a** 5D (HACA)CON(CA)CONH (N_i_–CO_i−1_ & N_i−1_–CO_i−2_) and **b** 5D HN(CA)CONH (HN_i_–N_i_ & HN_i+1_–N_i+1_)

**Fig. 3:**
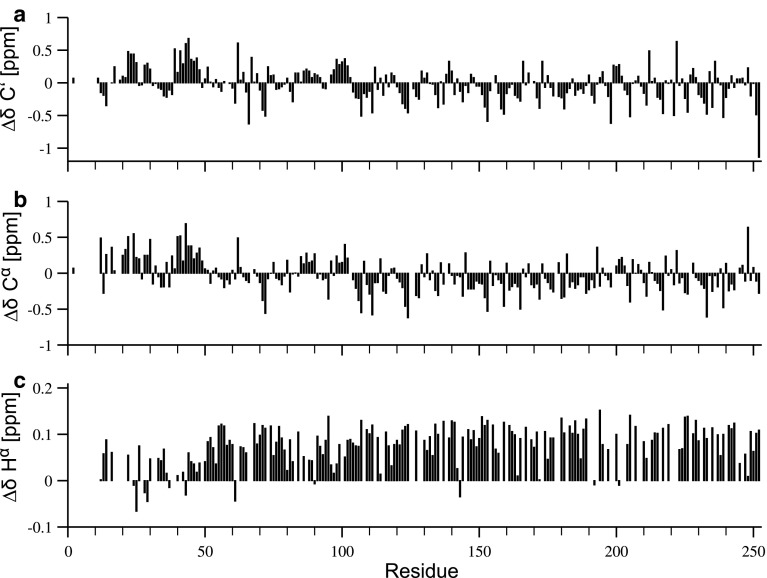
Secondary chemical shifts for **a** ^13^C′, **b** ^13^C^α^, and **c** ^1^H^α^ using sequence-specific random coil chemical shifts of intrinsically disordered proteins (Tamiola et al. 2010)

The ^1^H–^15^N HSQC spectrum of H6-hBASP1 shows a very narrow peak dispersion in the ^1^H dimension typical for intrinsically disordered proteins (Fig. 1). Extensive signal overlap in conventional 2D & 3D spectra could be overcome by using the aforementioned 5D experiments. 99 % of backbone ^15^N, 99.5 % of ^1^H^N^, 96.5 % of ^13^C^α^, 74 % of ^1^H^α^, 86.2 % of ^13^C^β^, 81.4 % of ^1^H^β^ and 98.7 % of ^13^C′ resonances have been assigned. Additionally, H(CC-tocsy)CONH spectra allowed the assignment of several side-chain atoms. Figure 2 shows sample strips of sequential resonance assignment in a 5D (HACA)CON(CA)CONH and HN(CA)CONH experiment. Secondary chemical shifts for ^13^C′, ^13^C^α^, ^1^H^α^ (Fig. 3) show only minor deviations from random coil chemical shift values. Interestingly, the N-terminus appears to harbour stretches with slight α-helical structure propensities, whereas the rest of the protein seems to adopt a rather extended conformation indicated by positive ^1^H^α^ chemical shift differences.

The ^1^H, ^13^C and ^15^N chemical shifts have been deposited in the BioMagResBank (http://www.bmrb.wisc.edu/) under the BMRB accession number 18417.
